# Home- vs gym-based exercise delivery modes of two multicomponent intensity training regimes on cardiorespiratory fitness and arterial stiffness in adults with intellectual and developmental disability during the COVID-19 pandemic – a randomized controlled trial

**DOI:** 10.1177/17446295241242507

**Published:** 2024-03-27

**Authors:** Xavier Melo, Bruno Simão, Catarina Catela, Isabel Oliveira, Sara Planche, Ana Louseiro, João Luís Marôco, Guillermo R Oviedo, Bo Fernhall, Helena Santa-Clara

**Affiliations:** Centro de Investigação Interdisciplinar Egas Moniz (CiiEM), 386312 Egas Moniz School of Health & Science, Monte da Caparica, Portugal; Centro Interdisciplinar de Estudo da Performance Humana (CIPER), 70882 Faculdade de Motricidade Humana – Universidade de Lisboa, Lisboa, Portugal; Centro Interdisciplinar de Estudo da Performance Humana (CIPER), 70882 Faculdade de Motricidade Humana – Universidade de Lisboa, Lisboa, Portugal; Research & Development Department, GCP Lab, Ginásio Clube Português, Lisboa, Portugal; Research & Development Department, GCP Lab, Ginásio Clube Português, Lisboa, Portugal; Faculdade de Ciências da Saúde e do Desporto, 126808 Universidade Europeia, Lisboa, Portugal; Research & Development Department, GCP Lab, Ginásio Clube Português, Lisboa, Portugal; Integrative Human Physiology Laboratory, Manning College of Nursing & Health Sciences, 14708 University of Massachusetts Boston, Boston, MA, USA; Faculty of Psychology, Education and Sport Science Blanquerna, 216341 University Ramon Llull, Barcelona, Spain; Integrative Human Physiology Laboratory, Manning College of Nursing & Health Sciences, 14708 University of Massachusetts Boston, Boston, MA, USA; Centro Interdisciplinar de Estudo da Performance Humana (CIPER), Faculdade de Motricidade Humana – Universidade de Lisboa, Lisboa, Portugal; Research & Development Department, GCP Lab, Ginásio Clube Português, Lisboa, Portugal

**Keywords:** intellectual developmental disability, covid-19, blood pressure, arterial stiffness, cardiorespiratory fitness, physical exercise, multicomponent exercise, delivery mode

## Abstract

**Background:** We compared the effects of home- vs gym-based delivery modes of two 8-week supervised multicomponent intensity training regimes on cardiorespiratory fitness and arterial stiffness in 17 adults with intellectual and developmental disability during the COVID-19 pandemic. **Methods**: Participants were assigned to sprint interval training or continuous aerobic training, both incorporating resistance training. The intervention started with 8-weeks of online training (M1-M2), 1-month of detraining, plus 8-weeks of gym-based training (M3-M4). **Results**: Peak oxygen uptake decreased from M1-M2 and increased from M2-M4. Central arterial stiffness decreased between M1-M2, and M1-M4, along with peripheral arterial stiffness. Central systolic blood pressure decreased from M1-M2 only with sprint interval training. **Conclusion**: Home-based training minimized the negative impact of the lockdown on central arterial stiffness and central blood pressure, but it did not match the benefits on cardiorespiratory fitness and peripheral arterial stiffness of a gym-based intervention, irrespective of the multicomponent intensity training regime. Registered in ClinicalTrials.gov NCT05701943.

## Introduction

The COVID-19 pandemic has cast a spotlight on the intensified challenges faced by individuals with deficits in intellectual and adaptive functioning in the conceptual, social and practical domain, referred to hereafter as intellectual and developmental disabilities (American Psychiatric Association, 2022), exacerbating their health issues, mental well-being, and societal disadvantages (Emerson and Hatton, 2008; Kirby, 2020; Qiao, 2020; WHO, 2020).

While the mandatory lockdowns have presented substantial hurdles for these individuals in maintaining physical activity levels (Fitzgerald et al., 2022), the aftermath of the pandemic brings to the forefront the persistent need for flexible and accessible exercise interventions (Theis et al., 2021). Beyond the immediate risk of infection, this population continues to grapple with multifaceted barriers that curtail their engagement in community-based exercise programs (Jacinto et al., 2021). It is evident that people with intellectual and developmental disabilities have long faced challenges in meeting physical activity requirements and maintaining optimal cardiorespiratory fitness (Gawlik et al., 2017; Hilgenkamp and Baynard, 2018). Existing research underscores their susceptibility to cardiovascular risk factors (Gawlik et al., 2017; Hsieh et al., 2014), highlighting the pressing need for effective interventions that can mitigate these risks. The increased vulnerability of this population to infection and COVID-19 complications is a paramount concern (Zbinden-Foncea et al., 2020), emphasizing the significance of measures that can both enhance cardiovascular well-being and reduce the risk of infectious diseases.

Prolonged periods of reduced physical activity, a byproduct of lockdowns, can lead to changes in the material properties of the arterial wall (Bohn et al., 2017). These alterations, in turn, have functional consequences for the artery, influencing the way pressure, blood flow, and arterial diameter change with each heartbeat (Salvi, 2016). This concept is known as arterial stiffness and can further amplify the risk of cardiovascular disease (Cavalcante et al., 2011; Hansen et al., 2006; Naqvi and Lee, 2014). Arterial stiffness can be measured in various types of arteries, including muscular and elastic ones, and assessed in cross-section, longitudinally along the vessel, or in both directions. Typically, arterial stiffness is evaluated by measuring the velocity of the pulse-wave travelling in a specific segment, commonly carotid-femoral pulse wave velocity (cfPWV). The pulse wave travels through the aorta, and its velocity is inversely related to the distensibility of the arterial wall. In simpler terms, the higher cfPWV, the lower the vascular distensibility, providing insights into the rigidity of the aorta. However, while the association of time spent in physical activities and sedentary behaviors with cfPWV has been studied in the general population (Germano-Soares et al., 2018), research in people with intellectual and developmental disabilities has remained limited, primarily focusing on individuals with Down syndrome (Rodrigues et al., 2011). This underscores the necessity for comprehensive investigations encompassing various etiologies within this diverse population.

Exercise training, whether aerobic-only (Boer et al., 2014; Melo et al., 2021) or multicomponent, also incorporating resistance training (Mendonca et al., 2011; Oviedo et al., 2014), improves cardiorespiratory fitness (2 to 6 mL.kg.min^-1^) and brachial systolic blood pressure (bSBP) (-15 to -6 mmHg) in people with intellectual and developmental disabilities. Specifically, Calders et al. (2011) demonstrated greater reductions in bSBP with a 12-week multicomponent training regime compared to an aerobic-only training group. Both the multicomponent and aerobic-only training groups (n = 15 each) exhibited similar increases in aerobic capacity. These findings collectively suggest that exercise training, particularly a multicomponent regime, is beneficial for people with intellectual and developmental disabilities. However, the nuanced effects of exercise intensity and its impact on arterial stiffness in this population remain ambiguous, as similar results have been reported with continuous aerobic training and high-intensity interval training for central and peripheral arterial stiffness (Kim, 2017; Melo et al., 2021). Importantly, these observations have predominantly stemmed from supervised gym or laboratory-based interventions, avenues that became largely inaccessible during the COVID-19 lockdown.

As we navigate the post-pandemic landscape, home-based exercise interventions takes on even greater significance. Challenges like travel time, financial constraints, and anxiety associated with novel environments have perennially hindered the involvement of people with intellectual and developmental disabilities in community-based programs (Jacinto et al., 2021). Online or virtual interventions hold promise in addressing these barriers and providing a more inclusive, accessible, and tailored approach to exercise engagement (Fjellstrom et al., 2022). This assumption is reinforced as the shadow of potential infectious disease outbreaks looms large (Smith, 2021), emphasizing the need for interventions that ensure sustained and consistent access to exercise.

Hence, in view of these complex and evolving considerations, this study endeavored to compare the effectiveness of two distinct 8-week supervised multicomponent intensity training regimes, delivered either at home or at the gym, in improving cardiorespiratory fitness and managing arterial stiffness in people with intellectual and developmental disabilities. We hypothesized that home- and gym-based exercise delivery modes hold equal potential in augmenting cardiorespiratory fitness and overall arterial health in this population, irrespective of the multicomponent intensity training regimes.

## Methodology

### Design

**Figure 1.**
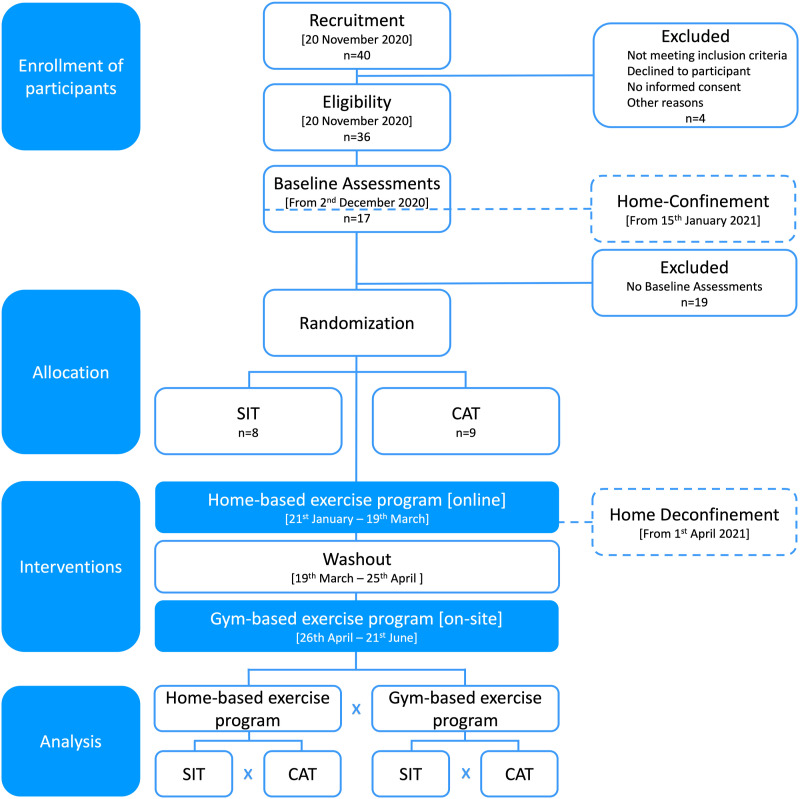
Participant flow diagram. *Abbreviations: SIT = sprint interval training; CAT = continuous aerobic exercise training.*

This study was a 16-week parallel group randomized controlled trial in which participants were randomly assigned in a 1:1 allocation ratio to a multicomponent exercise training regime, either with sprint interval training or continuous aerobic training. Randomization was done using a random-block scheme obtained from https://www.randomizer.org/. Both groups exercised for 60 minutes, 3 times per week. The intervention started with 8 weeks of online training via Google Meets at their homes, followed by 1 month of detraining with no exercise besides those available by the SPORTS4ALL program, and another 8 weeks of on-site training at the gym in GCP. Participants' exercise adherence was evaluated using an exercise diary maintained by the exercise physiologists. The study timeline, intervention, and assessments are presented in Figure 1, and all related trials were retrospectively registered on ClinicalTrials.gov under NCT05701943 due to human, time, and logistical constraints faced during the COVID-19 pandemic. The primary outcomes were cardiorespiratory fitness (peak VO_2_) and central arterial stiffness (cfPWV), assessed at GCP laboratory on 4 occasions over a 20-week period: before (M1) and after (M2) the home-based intervention, and before (M3) and after (M4) the gym-based intervention. All laboratory tests were conducted by the same researchers at a room temperature of 22–24°C, in the morning, after a fasting period of 3 hours, no alcohol for 24 hours, no caffeine for 8 hours, and no vigorous exercise for 48 hours before data collection. On the day of testing, participants had their body composition measured, brachial arterial pressure assessed, arterial stiffness evaluated by applanation tonometry, and completed an incremental test to exhaustion.

### Participants

Based upon a small effect size of 0.173 derived from a target increase of 0.3 L.min-1 and a SD of 0.4 L.min-1 in peak VO_2_ (Boer and Moss, 2016), 15 participants were required per group, assuming a 95% confidence level, 5% alpha error, and 20% beta error, with a 1:1 ratio between groups. To account for a 20% dropout rate, a total of 36 participants (18 per group) were recruited from the social project SPORTS4ALL by Ginásio Clube Português (GCP). These people have any type of disability or limitation, and the project ensures equality of opportunities in well-being, leisure, and culture through sports. Study advertising took place through invitation by the research team. Thirty-six participants met the following inclusion criteria: ≥ 18 and ≤ 55 years; diagnosed with mild to moderate intellectual and developmental disabilities; exercised at least 1 d/week in the last 2 months; able to participate in group exercise activities with ≥ 8 people; able to walk independently; and able to perform all physical fitness assessments. Four participants were excluded because they had 1 of the following: cardiovascular disease, respiratory disorder, metabolic disease, atlantoaxial instability, severe intellectual and developmental disabilities, smoking, and/or use of heart rate and blood pressure altering or non-steroidal anti-inflammatory medications, inability to comply with guidelines for participation in exercise testing and training (Riebe et al., 2018). Informed written consent was signed by all participants and parent(s) and legal guardian(s), and participants or parents/legal guardians filled-out the Physical Activity Readiness Questionnaire for Everyone, and a sociodemographic questionnaire. By the time the mandatory COVID-19 home confinement was declared by the Portuguese government, only 17 participants had completed laboratory exercise testing. Only these participants took part in this study. This study was approved by the Ethical Committee of Faculdade de Motricidade Humana – Universidade de Lisboa (46/2021).

### Exercise interventions

The study implemented exercise programs for a duration of 8 weeks, which were delivered to participants in their homes via Google Meets, as well as at the gym. The sessions were designed by exercise physiologists and rehabilitation technicians in adherence to the American College of Sports Medicine (ACSM) guidelines (Riebe et al., 2018) and the National Strength and Conditioning Association (NSCA) (Haff et al., 2016). Exercise physiologists underwent standardized training and a 2-month familiarization period. Both multicomponent intensity training regimes comprising aerobic, resistance, balance, and flexibility exercises, were divided into 2 groups, each with 8-9 participants, maintaining a participant-exercise physiologist ratio of 3:1. In each exercise session, whether home- or gym-based, at least one rehabilitation technician was also present. Both programs were divided into three phases, which were adapted from Oviedo et al., (2020): the initial phase (weeks 1-3), the improvement phase (weeks 4-6), and the maintenance phase (weeks 7-8). The multicomponent intensity training regimes were matched for total duration, and all sessions included a standardized 5-minute warm-up and cool-down exercises. During the washout period, 14 participants underwent 3 sessions, each corresponding to a phase of the home-based intervention. These sessions were conducted while participants wore heart rate monitors, providing a proxy for exercise intensity during each phase of the home-based intervention. Both multicomponent intensity training regimes comprised a standard resistance training program, consisting of 6 exercises targeting various muscle groups. For the upper limbs, these exercises comprised seated biceps curls, triceps extensions, and frontal shoulder raises. For the torso, seated rows and side bends were included, while chair squats were performed for the lower limbs. All exercises were executed using either 2-4 kg dumbbells or the participants' own body weight, based on individual capabilities. To monitor resistance training intensity, the 0-10 points Rate of Perceived Exertion OMNI-Resistance Exercise Scale (RPE OMNI-RES) (Gearhart et al., 2009) was employed. The intensity was kept constant at 2 x 12 repetitions at RPE OMNI-RES 8 throughout the study.

#### Multicomponent continuous aerobic training regime

The multicomponent continuous aerobic training regime, henceforth referred to as continuous aerobic training, encompassed a comprehensive set of exercises, incorporating aerobic, resistance, balance, and flexibility exercises (see Supplement 1). For the aerobic component, participants engaged in moderate intensity, steady-state exercises for an extended period, tailored to their intervention location. In the home-based intervention, participants engaged in bodyweight exercises, such as low-impact jumping jacks, slow standing box, slow side shift with floor touch, slow high knees, half burpee (without jump), and hook box (Borrega-Mouquinho et al., 2021). In the gym-based intervention, participants used cycle ergometers (Star Trac Spinner Blade ION 7220, Vancouver, WA) for their aerobic component. Each session began with a 5-minute warm-up, followed by three continuous cycling bouts at a steady-state intensity. The duration of each bout (5-10 minutes) and the intensity (55-85% heart rate reserve) were progressively increased in each phase. Participants exercised at the target intensity using the OMNI scale 4-6 (Stanish and Aucoin, 2007) during home-based intervention and an heart rate chest band (H10 Polar, Electro, Kempele, Finland) during gym-based intervention.

#### Multicomponent sprint interval training regime

The multicomponent sprint interval training regime, henceforth referred to as “sprint interval training”, a form of high intensity interval training, also encompassed a comprehensive set of exercises, incorporating aerobic, resistance, balance, and flexibility exercises (Supplement 2). However, the aerobic component featured short bursts of high-intensity sprints followed by periods of lower-intensity activity or rest, performed differently depending on the location of the intervention. In the home-based intervention, bodyweight exercises such as jumping jacks, standing box, side shift with floor touch, high knees, half burpee, and hook box were used (Borrega-Mouquinho et al., 2021). In the gym-based intervention, cycle ergometers (Star Trac Spinner Blade ION 7220, Vancouver, WA) were utilized instead. The aerobic component began with a 5-minute warm-up, followed by bouts of 5-10 minutes of exercise, consisting of 5-20 second all-out sprints followed by 15-45 seconds of low cadence recovery (1:3-1:2 work-rest ratio). The duration of sprints and active recovery were adjusted throughout the program. The OMNI scale 8-10 (Stanish and Aucoin, 2007) was used to ensure that participants exercised at the target intensity during the home intervention, and an heart rate chest band (H10 Polar, Electro, Kempele, Finland) was used during the gym intervention.

### Cardiopulmonary exercise test

Participants completed a ramp incremental cycle ergometer test to exhaustion on a calibrated electronically braked cycle ergometer (Monark 839 E, Ergomedic; Monark, Vansbro, Sweden) at a pedal cadence of 70-75 rev.min^-1^ with initial and incremental workloads of 10-20 watts. Respiratory gases were continuously analyzed, with a mixing-chamber method using a portable gas analyzer (K5, Cosmed, Rome, Italy), calibrated according to the manufacturer’s instructions. HR was monitored continuously (Garmin, US) and data were evaluated in 10-s averages. Peak VO_2_ was defined as the highest 20-s value attained in the last minute of effort when 2 of the following criteria were met: (1) ∼90% of predicted maximal HR: 210 - 0.56 (age) - 15.5 (Down Syndrome) (Fernhall et al., 2001); (2) Plateau in VO_2_ with an increase in workload (<2.0 mL.kg^-1^.min^-1^); (3) Respiratory exchange ratio ≥ 1.1; and/or (4) subjective judgment that the participant could no longer continue. Results were compared to age and sex normative data (Pescatello et al., 2014). Chronotropic responses were calculated as HR reserve/ (predicted/maximal HR - HR at rest)*100 (Azarbal et al., 2004). Chronotropic incompetence was defined as the failure to reach 80% of the chronotropic response (Brubaker and Kitzman, 2011). HR recovery was calculated as the difference in heart rate after 1-min of recovery in relation to the peak heart rate. An abnormal heart rate recovery was defined as reductions of less than 12 b.min^-1^ (Cole et al., 1999). The first ventilatory threshold (VT1) was identified as 1) the first increase in the ventilatory equivalent for O_2_ (VE/VO_2_), without any increases in the ventilatory equivalent for CO_2_ (VE/VCO_2_), and 2) the first increase in the expiratory fraction of O_2_. The first increase in the ventilatory equivalent for CO2 (VE/VCO_2_) and the first reduction in the expiratory fraction of CO_2_ were identified as the second ventilatory threshold (VT2) (Meyer et al., 2005).

### Regional arterial stiffness and blood pressure

The study utilized piezoelectric pressure mechanotransducers to measure arterial stiffness through PWV (Complior Analyse, ALAM Medical, Paris, France). The pressure waveform travel distance (d) was measured between the carotid-femoral (cf), and carotid-distal posterior tibial arteries (cd) using tape measures, and the Pulse transit time (PTT) was calculated using the intersect tangent algorithm of the foot-to-foot method. The measurements were taken by a single operator on the right side of the body after 10 high quality pulse waveforms (>90%). The cfPWV and cdPWV were used as indices of central and peripheral arterial stiffness, respectively (Salvi et al., 2019; Townsend et al., 2015). The central SBP (cSBP) was assessed using the piezoelectric pressure mechanotransducers placed in the carotid artery, calibrated from the brachial mean arterial pressure (bMAP), and the mean values were extracted from a 15-s window of acquisition. The cfPWV was compared to established European reference values (The Reference Values for Arterial Stiffness’ Collaboration, 2010a). The inter-day CV for cfPWV and cdPWV were 1.12% and 3.02%, respectively.

### Body composition

Height, waist circumference, and weight were measured to the nearest 0.1 cm and nearest 0.1 kg, respectively, on a scale with an attached stadiometer (model 770, Seca; Hamburg, Deutschland). Body composition was measured using a seca mBCA 515 using 8 electrodes.

### Statistical analyses

All statistical analyzes were conducted with a significant level (α) of 0.05 using the R software, version 4.1.2. We performed an intention-to-treat analysis using all randomized participants. The data are presented as mean and standard deviation. To compare baseline characteristics Welch’s independent t-tests were used.

Changes in the incidence of participants with low cardiorespiratory fitness, high bSBP, cfPWV, and BMI throughout the study period were tested using generalized estimating equation models. The models were fitted following a Poisson distribution using the geepack package (Halekoh et al., 2006).

The changes in the cardiorespiratory fitness and vascular outcomes were examined using linear mixed models fitted with restricted maximum likelihood and applying Satterthwaite's method for approximating degrees of freedom for the F test from the R lmerTest package (Kuznetsova et al., 2017). The fixed effects were time (home- and gym-based) and multicomponent intensity training regime (continous aerobic training and sprint interval training), whereas the random intercept was defined for each participant. All models’ residuals were tested for normality and homogeneity of variances using both Shapiro-Wilk and  Breusch-Pagan tests, respectively, and via QQ plot inspection using the R performance package (Lüdecke, Makowski, et al., 2020b) Using the R sjstats package (Lüdecke, Ben-Shachar, et al., 2020a), partial eta squares (η^2^) were calculated for main effects and interaction and interpreted using the benchmarks suggested by Cohen (1988) defining small (η^2^ < 0.05), medium (η^2^ < 0.25), and large (η^2^ > 0.25) effect sizes. In the presence of significant main effects and interactions, post-hoc comparisons using Tukey's HSD test were conducted using the R emmeans package (Lenth R, 2020).

## Results

### Characterization of the exercise training programs

The baseline characteristics of the participants are presented in Table 1. The home-based intervention attendance rate was 81% (sprint interval training = 92%; continuous aerobic training = 69%) with a mean of 19 (sprint interval training = 22 sessions; continuous aerobic training = 17 sessions) out of 24 training sessions offered. Four participants in continuous aerobic training and 1 participant in sprint interval training attended less than 75% of the home-based sessions. The gym-based intervention attendance rate was 79% (sprint interval training = 77%; continuous aerobic training = 80%) with a mean of 19 (sprint interval training = 19; continuous aerobic training = 19) out of 24 training sessions offered. Three participants in sprint interval training attended less than 75% of the gym-based intervention (38%, 63%, and 71%). Reasons behind the insufficient attendance rates included illness (n = 1), schedule mismatch (n = 5), and technical issues during the home-based sessions (n = 2). No instances of harm, adverse events, or SARS-CoV-2 virus infections were reported throughout the duration of the study.

**Table 1.** Baseline characteristics of the participants by multicomponent intensity training regime.

Variables	SIT (n=8)	CAT (n=9)	(*p*-value)
Characteristics			
Age (years)	26±8	29±13	*p* = 0.46
Sex (male/female)	6/2	5/4	*p* = 0.43
Resting heart rate (b.min^-1^)	80±11	79±14	*p* = 0.88
Etiology			
*Autism spectrum disorder*	3	0	*p* = 0.04
Down Syndrome	2	3	*p* = 0.33
Global Development Delay	1	4	*p* = 0.17
Intellectual Disability	2	1	*p* = 0.48
Williams Syndrome	0	1	*p* = 0.36

Data are presented as mean ± standard deviation. Abbreviations: SIT = sprint interval training; CAT = continuous aerobic exercise training.

Home-based and gym-based exercise physiological demands are depicted in Supplement 4. Mean heart rate (difference (d) = 15, 95% CI: -1 to 31 b.min^-1^, *p* = 0.06) and %heart rate reserve (d = 2, 95% CI: -6 to 10 b.min^-1^, *p* = 0.54) during sprint interval training and continuous aerobic training in the home-based intervention did not differ. In the gym-based intervention, the mean heart rate (d = 25 b.min^-1^, 95% CI: 9 to 40 b.min^-1^, *p* = 0.01), but not the mean %heart rate reserve (d = 5, 95% CI: -3 to 13 b.min^-1^, *p* = 0.17), was higher during sprint interval training compared to continuous aerobic training, respectively. There were no differences in heart rate and %heart rate reserve between exercise delivery modes (Supplement 3)

### Cardiorespiratory fitness

**Figure 2.**
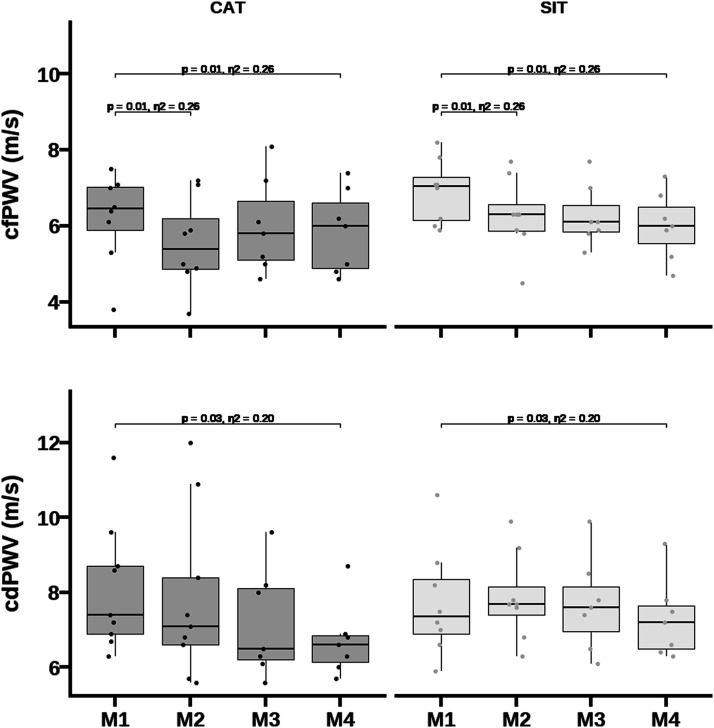
Comparison of central (cfPWV) and peripheral arterial (cdPWV) stiffness among delivery exercise modes and multicomponent intensity training regimes.

No baseline differences were observed between multicomponent intensity training regimes in cardiorespiratory fitness indices (Table 2). All participants remained classified as unfit from M1 to M4. Abnormal heart rate recovery and chronotropic incompetence were present in 24% and 18% of the participants, respectively. A main effect of time was observed for peak VO_2_ [F (3,39) = 4.68; *p *= 0.007; η^2^ = 0.26)], suggesting a decrease from M1 to M2 (d= -1.8, 95% CI: -0.2 to -3.4 mL.kg.min^-1^, *p* = 0.02), and an increase from M2 to M4 (d = 2.1, 95% CI: 0.4 to 3.8 mL/kg/min, *p *= 0.01; Figure 2), independent of age, sex, and fat mass. Main effects of time were also observed for power output [F (3,37) = 5.07; *p *= 0.005; η^2^ = 0.29)] and for time-to-exhaustion [F (3,38) = 3.37; *p* = 0.03; η^2^ = 0.21)]. These suggested decreases from M1 to M2 (d = -27, 95% CI: -49 to 5 W, *p* = 0.01), but increases (d = 26, 95% CI: 3 to 50, *p* = 0.02) from M2 to M4 in power output, together with CPET duration (d = 104, 95% CI: 14 to 194 s, *p* = 0.02). A multicomponent intensity training regime-by-time interaction effect was observed for VT2 [F (3,39) = 3.93; *p* = 0.02; η^2^ = 0.23)], suggesting that VT2 decreased from M1 to M2 in sprint interval training (d = -5.2, 95% CI: -8.8 to -1.6 mL.kg.min^-1^, *p*< 0.01), and from M2 to M3, M2 to M4 in continuous aerobic training (d = 3.8, 95% CI: 0.1 to 7.6 mL/kg/min, *p* = 0.04 and d = 4.7, 95% CI: 0.9 to 8.4 mL.kg.min^-1^, *p* = 0.01, respectively), independent of age, sex, and peak VO_2_.

**Table 2.** Comparison of indices of cardiorespiratory fitness among delivery exercise modes and multicomponent intensity training regimes.

Cardiorespiratory fitness		SIT	CAT	Main effect of time (p-value; partial eta square)	Main effect of multicomponent intensity training regimes (p-value; partial eta square)	Interaction (p-value; partial eta square)
Time to peak power (s)				(*p* = 0.02; *η^2^*= 0.21)	(*p* = 0.19; *η^2^*= 0.13)	(*p* =0.98; *η^2^*= 0)
	**M1**	557±132	625±193	-	-	-
	**M2**	497±75	553±92	M2 < M4	-	-
	**M3**	540±79	623±90	-	-	-
	**M4**	595±176	663±72	-	-	-
Peak Power (W)				(*p* < 0.01; *η^2^* = 0.29)	(*p* = 0.71; *η^2^*= 0.00)	(*p* = 0.71; *η^2^* = 0.04)
	**M1**	205±25	192±39	M1 > M2	-	-
	**M2**	168±25	174±26	M2 < M4	-	-
	**M3**	184±20	177±35	-	-	-
	**M4**	200±24	195±45	-	-	-
Heart rate max (b.min^-1^)				(*p* = 0.03; *η^2^*= 0.19)	(*p* = 0.10; *η^2^*= 0.17)	(*p* = 0.44; *η^2^* = 0.07)
	**M1**	159±13	151±29	-	-	-
	**M2**	158±17	143±26	-	-	-
	**M3**	146±21	139±19	-	-	-
	**M4**	143±19	143±24	-	-	-
RER				(*p* = 0.18; *η^2^*= 0.12)	(*p* = 0.10; *η^2^*= 0.17)	(*p* = 0.46; *η^2^* = 0.06)
	**M1**	1.16±0.14	1.00±0.10	-	-	-
	**M2**	1.11±0.16	1.07±0.10	-	-	-
	**M3**	1.11±0.14	1.05±0.09	-	-	-
	**M4**	1.12±0.18	1.00±0.10	-	-	-
Heart rate recovery 1^st^ Minute (b.min^-1^)				(*p* = 0.48; *η^2^*= 0.06)	(*p* = 0.83; *η^2^*= 0.00)	(*p* = 0.57; *η^2^*= 0.05)
	**M1**	19±11	18±6	-	-	-
	**M2**	21±8	24±8	-	-	-
	**M3**	22±8	18±5	-	-	-
	**M4**	22±9	20±11	-	-	-
VT1 (mL.kg.min^-1^)				(*p* = 0.06; *η^2^*= 0.17)	(*p* = 0.23;*η^2^*= 0.10)	(*p* = 0.52; *η^2^*= 0.05)
	**M1**	16.8±5.0	13.7±5.0	-	-	-
	**M2**	12.7±4.7	10.4±6.0	-	-	-
	**M3**	16.2±9.0	10.8±2.0	-	-	-
	**M4**	15.5±6.2	15.1±5.0	-	-	-
VT2 ((mL.kg.min-1))				(*p* < 0.01; *η^2^*= 0.31)	(*p* = 0.19; *η^2^*= 0.11)	(*p* = 0.02; *η^2^*= 0.23)
	**M1**	26.2±6.2	18.6±6.5	M1 > M2	-	SIT M1 > SIT M2
	**M2**	21.0±5.7	16.4±5.1	-	-	-
	**M3**	23.1±6.6	19.7±7.2	M3 > M2	-	CAT M3 > CAT M2
	**M4**	23.0±6.0	20.5±5.5	M4 > M2	-	CAT M4 > CAT M2

Data are presented as mean ± standard deviation. Abbreviations: RER = respiratory exchange ratio; VT1 = first ventilatory threshold; VT2 = second ventilatory threshold; SIT = sprint interval training; CAT = continuous aerobic exercise training; M = moment.

### Arterial stiffness

No baseline differences were observed between multicomponent intensity training regimes in arterial stiffness indices (Figure 2). A main effect of time was observed in the incidence of participants with cfPWV above +2SD of the age- and blood pressure-specific mean reference values (χ^2^ = 9617, *p <* 0.001). At baseline, 24% of participants exhibited high central arterial stiffness, at M2 only 12% (*p* = 0.383), at M3 only 7% (*p* = 0.260) and at M4 (*p* < 0.001) none of the participants.

**Figure 3.**
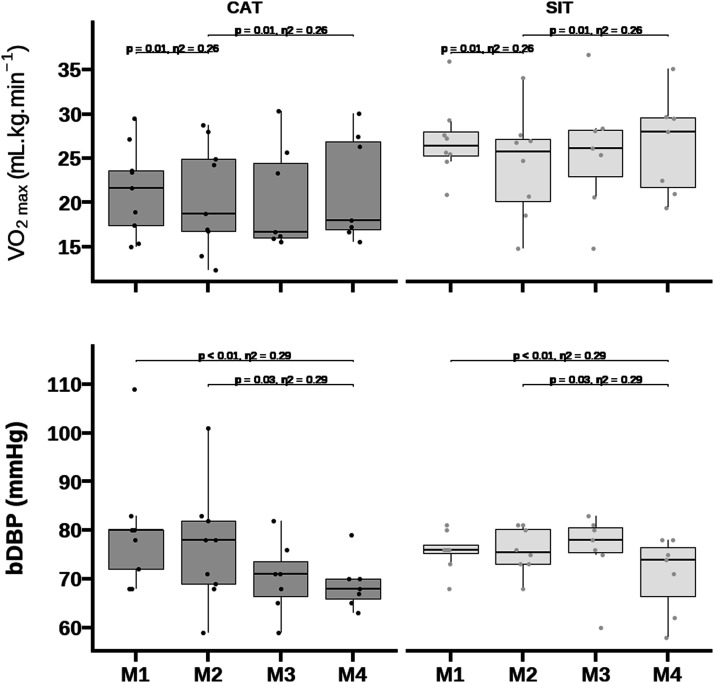
Comparison of peak oxygen uptake and diastolic blood pressure among delivery exercise modes and multicomponent intensity training regimes.

Main effects of time were observed for cfPWV [F (3,38)= 5.15; *p* = 0.004; η^2^ = 0.29)] and for cdPWV [F (3,39)= 3.32; *p* = 0.03; η^2^ = 0.29)]. These suggested a reduction in cfPWV from M1 to M2 (d = -0.61, 95% CI: -1.1 to -0.1 m.s^-1^ *p* = 0.01), and in both cfPWV (d = -0.63, 95% CI: -1.1 to -0.1 m.s^-1,^ *p* = 0.01) and cdPWV (d = -0.72, 95% CI: -1.4 to -0.1 m/s, *p* = 0.03; Figure 3) from M1 to M4. Changes in cfPWV, but not in cdPWV, remained significant after adjustment for bMAP, peak VO_2_, height, fat mass, resting heart rate, and age.

### Blood pressure

No baseline differences were observed between multicomponent intensity training regimes in hemodynamic parameters (Table 3). The incidence of elevated bSBP (Casey et al., 2019) was 65% at M1, 47% at M2, 35% at M3, and 35% at M4 but these changes did not reach statistical significance (χ^2^ = 2.40, *p =* 0.49). Main effects of time were observed for bDBP [F (3,39) = 5.32; *p* = 0.004; η^2^ = 0.29), Figure 1] and bMAP [F (3,39) = 5.28; *p* = 0.004; η^2^ = 0.29)]. These supported a decrease from M1 to M4 (bDBP: d = -6, 95% CI: -10 to -2 mmHg, *p* = 0.002; bMAP: d = -6, 95% CI: -10 to -2 mmHg, *p* = 0.003]), from M2 and M4 (bDBP: d = -5, 95% CI: -9 to -1 mmHg, *p* = 0.03; bMAP: d = -4, 95% CI: -9 to -1 mmHg, *p* = 0.03), independently of age, sex, cfPWV, peak VO_2_, and fat mass. A multicomponent intensity training regime-by-time interaction effect was observed for cSBP [F (3,39) = 2.85; *p* = 0.04; η^2^ = 0.18)], suggesting a decrease from M1 to M2 in sprint interval training (d = -17, 95 to -1 mmHg, *p* = 0.03), independently of age, sex, and peak VO_2_, but not cfPWV and fat mass.

**Table 3.** Comparison of hemodynamics indices among delivery exercise modes and multicomponent intensity training regimes.

Hemodynamics		SIT	CAT	Main effect of time (p-value; partial eta square)	Main effect of multicomponent intensity training regimes (p-value; partial eta square)	Interaction (p-value; partial eta square)
bSBP (mmHg)				(*p* = 0.23; *η^2^* = 0.11)	(*p* = 0.81; *η^2^* = 0.00)	(*p* = 0.19; *η^2^* = 0.11)
	**M1**	125±13	122±19	-	-	-
	**M2**	122±12	124±18	-	-	-
	**M3**	120±13	121±11	-	-	-
	**M4**	115±12	123±7	-	-	-
bDBP (mmHg)				(*p* < 0.01; *η^2^* = 0.29)	(*p* = 0.82; *η^2^* = 0.00)	(*p* = 0.28; *η^2^* = 0.09)
	**M1**	76±4	80±12	M1 > M4	-	-
	**M2**	80±5	77±12	M2 > M4	-	-
	**M3**	76±8	74±7	-	-	-
	**M4**	71±8	72±5	-	-	-
bMAP (mmHg)				(*p* < 0.01; *η^2^* = 0.29)	(*p* = 0.80; *η^2^* = 0.00)	(*p* = 0.45; *η^2^* = 0.06)
	**M1**	92±5	94±13	M1 > M4	-	-
	**M2**	91±7	92±13	M2 > M4	-	-
	**M3**	91±8	89±7	-	-	-
	**M4**	85±9	89±5	-	-	-
bPP (mmHg)				(*p* = 0.87; *η^2^* = 0.02)	(*p* = 0.94; *η^2^* = 0.00)	(*p* = 0.09; *η^2^* = 0.15)
	**M1**	49±13	42±14	-	-	-
	**M2**	46±9	47±13	-	-	-
	**M3**	44±11	46±11	-	-	-
	**M4**	45±8	50±7	-	-	-
cSBP (mmHg)				(*p* = 0.36; *η^2^* = 0.08)	(*p* = 0.39; *η^2^* = 0.05)	(*p* = 0.04; *η^2^* = 0.18)
	**M1**	140±30	118±20	-	-	SIT M1 > SIT M2
	**M2**	123±21	123±15	-	-	-
	**M3**	131±30	121±12	-	-	-
	**M4**	124±32	120±8	-	-	-

Data are presented as mean ± standard deviation. Abbreviations: bSBP = brachial systolic blood pressure; bDBP = brachial diastolic blood pressure; bMAP = brachial mean arterial pressure; bPP = brachial pulse pressure; cSBP = central systolic blood pressure; SIT = sprint interval training; CAT = continuous aerobic exercise training; M = moment.

### Body composition

No baseline differences were observed between multicomponent intensity training regimes in body composition outcomes at M1. At baseline, 41% of the participants were overweight and 12% obese, while at M3, 24% were overweight and 12% obese (χ^2^ = 1.08 *p=* 0.78) based on body mass index (BMI). Increased central fat accumulation was observed in 65% of the participants at M1, and 50% at M3. A main effect of time was observed for %fat mass [F (3,38) = 3.76; *p*= 0.02; η^2^= 0.23)], suggesting a decrease from M2 to M4 (d= -2.0, 95% CI: -3.0 to -0.2%, *p*= 0.01), independently of age and sex (Supplement 4)

## Discussion

The study found that home and gym-based exercise are equally effective at reducing central arterial stiffness, but only gym-based exercise led to improvements in peripheral arterial stiffness, cardiorespiratory fitness, bDBP, and fat mass. Both multicomponent intensity training regimes increased cardiorespiratory fitness and reduced central and peripheral arterial stiffness, but only sprint interval training reduced cSBP. Thus, while the home-based intervention may have mitigated COVID-19's harmful effects on arterial health, gym-based exercise provided greater cardiovascular benefits for people with intellectual and developmental disabilities, regardless of the multicomponent intensity training regime.

### Cardiorespiratory fitness

Aerobic training improves cardiorespiratory fitness in adults with intellectual and developmental disabilities (Boer et al., 2014; Boer and Moss, 2016; Melo et al., 2021). High-intensity aerobic exercise is more effective in improving cardiorespiratory fitness than low-intensity exercise when the volume is controlled (Martin-Smith et al., 2020). Sprint interval training yielded greater gain magnitude compared to continuous aerobic training in adolescents and young adults with intellectual and developmental disabilities (Boer et al., 2014) and adding 3 months of HIIT to 12 months of continuous aerobic training reduced the cardiorespiratory fitness non-response rate from 60% to 20% in adults with intellectual and developmental disease (Melo et al., 2021). In the present study, sprint interval training and continuous aerobic training increased peak VO_2_ comparably, consistent with previous reports (Su et al., 2019).

Our home-based intervention did not mitigate the negative impact of the 8-week mandatory home confinement on cardiorespiratory fitness. Peak VO_2_ typically experiences a 7-15% reduction after 2 weeks of bed rest (Pišot et al., 2016; Schwendinger and Pocecco, 2020). Our study observed similar decreases in peak VO_2_ over the home-confinement period, potentially increasing the risk of cardiovascular disease and mortality (Kodama et al., 2009). Gym-based exercise resulted in an 8% increase in peak VO_2_, possibly reducing all-cause mortality risk (Lee et al., 2011). High cardiorespiratory fitness is associated with lower risk of severe COVID-19 and related complications such as hospitalization, intensive care, and death (Af Geijerstam et al., 2021; Christensen et al., 2021; Ekblom-Bak et al., 2021; Ritter and Kararigas, 2021). Fat mass reductions noted only after gym-based training may have contributed to increases in cardiorespiratory fitness, suggesting that gym-based training induces greater energy expenditure compared to home-based training.

### Blood pressure and arterial stiffness

The effects of aerobic training on blood pressure (BP) and arterial stiffness in people with and without intellectual and developmental disabilities depend on exercise intensity and duration, with larger reductions observed in those with higher BP and stiffer arteries (Ashor et al., 2014; Huang et al., 2016; Zhang et al., 2018). However, the effectiveness of the exercise intervention appears to vary, as both sprint interval training and continuous aerobic training were equally effective in reducing regional stiffness and DBP, as reported in other studies (Hasegawa et al., 2018; Way et al., 2019). The discrepancies in findings may relate to differences in exercise intervention duration and intensity between studies. Both home- and gym-based exercise modes were effective in reducing central arterial stiffness, but decreases in peripheral arterial stiffness were only evident after the gym-based intervention. This suggests that remote multicomponent intensity training regimes using body weight, even without objective control of the intensity, are effective at controlling traditional cardiovascular risk factors and delivering vascular benefits. The findings also agree with those indicating that peripheral arterial stiffness is less amenable to change in short-exercise training interventions (Hayashi et al., 2005). Changes in central and peripheral arterial stiffness were associated with changes in MAP, confirming high BP as the main determinant of stiffer arteries through the promotion of matrix remodeling (Safar et al., 2018). However, the gym-based intervention led to a decrease in bDBP, suggesting that other mechanisms contribute to reductions in central arterial stiffness, including reductions in oxidative stress. Importantly, this exercise-dependent diastolic antihypertensive benefit is clinically relevant (> 5 mmHg), but the home-based intervention did not produce the same benefit. Nonetheless, the home-based intervention, particularly sprint interval training, reduced cSBP, an important predictor of cardiovascular disease (Lamarche et al., 2021).

### Limitations

This study is not without limitations. Our statistical inferences may be underpowered because we only sampled half of the intended participants due to COVID-19 constraints. However, we would like to emphasize that our conclusions are supported by large effect sizes observed, as reported for the main effects of time and multicomponent intensity training regimes -by-time interaction effects across various parameters. Still, readers should interpret these findings as preliminary. We used a cycle ergometer to measure cardiorespiratory fitness, which may not translate to other activities commonly prescribed to this population. Exercise testing validity and reliability, particularly with cycle ergometry, have been questioned in this population, but most CPETs in sprint interval training were maximal. However, only six CPETs were maximal in the continuous aerobic training. We could not objectively monitor exercise intensity during the home intervention because we did not have the means or authorization to deliver heart rate monitors to participants at home during the lockdown. Participants in the sprint interval training group did not achieve the target intensity in both delivery modes. The training regimes were not isocaloric, making effective comparisons challenging. We did not control dietary intake, which likely increased during the lockdown and could have affected study outcomes. Finally, our study included persons with intellectual and developmental disabilities who were familiar with exercise training, which limits the generalization of our findings to the sedentary population.

### Practical implications

The study's practical implications hold substantial significance for practitioners and policymakers directing their focus toward cardiovascular health in people with intellectual and developmental disabilities. The findings underscore the critical importance of tailoring exercise interventions to individual needs, with a noteworthy emphasis on the pivotal influence of the delivery mode over the multicomponent intensity training regimes. Gym-based exercises exhibited superior outcomes, affirming their effectiveness. However, the study also accentuates the value of adaptable home-based interventions, particularly in mitigating challenges such as central arterial stiffness during periods like lockdowns. These insights advocate for a balanced approach, urging practitioners to consider both home and gym-based strategies for the development of personalized and inclusive exercise programs, irrespective of the multicomponent intensity training regime. Policymakers can leverage this information to shape inclusive fitness policies, ensuring access to suitable facilities and fostering awareness of cardiovascular health within this population.

## Conclusion

A home-based exercise intervention minimized the negative physiological impact of a mandatory lockdown on central arterial stiffness and blood pressure but does not match the benefits in cardiorespiratory fitness, peripheral arterial stiffness, and fat mass of an iso-temporal gym-based intervention, irrespective of the multicomponent intensity training regime.

## Supplemental Material

Supplemental Material - Home- vs gym-based exercise delivery modes of two multicomponent intensity training regimes on cardiorespiratory fitness and arterial stiffness in adults with intellectual and developmental disability during the COVID-19 pandemic – a randomized controlled trial Supplemental Material for Home- vs gym-based exercise delivery modes of two multicomponent intensity training regimes on cardiorespiratory fitness and arterial stiffness in adults with intellectual and developmental disability during the COVID-19 pandemic – a randomized controlled trial by Xavier Melo, Bruno Simão, Catarina Catela, Isabel Oliveira, Sara Planche, Ana Louseiro, João Luís Marôco, Guillermo R Oviedo, Bo Fernhall, Helena Santa-Clara in Journal of Intellectual Disabilities

## Data Availability

The data set associated with this manuscript is deposited here: 10.17632/93w6dmhptd.1
