# Supplementary material for: Home- vs gym-based exercise delivery modes of two multicomponent intensity training regimes on cardiorespiratory fitness and arterial stiffness in adults with intellectual and developmental disability during the COVID-19 pandemic – a randomized controlled trial
Source: J Intellect Disabil. 2024 Mar 27;29(1):66–85. doi: 10.1177/17446295241242507 (PMC11898390; doi:10.1177/17446295241242507)
Supplement: Supplemental Material - Home- vs gym-based exercise delivery modes of two multicomponent intensity training regimes on cardiorespiratory fitness and arterial stiffness in adults with intellectual and developmental disability during the COVID-19 pandemic – a randomized controlled trial [file sj-pdf-1-jld-10.1177_17446295241242507.pdf]

Supplements

Supplement 1: Example of a multicomponent continuous aerobic training session

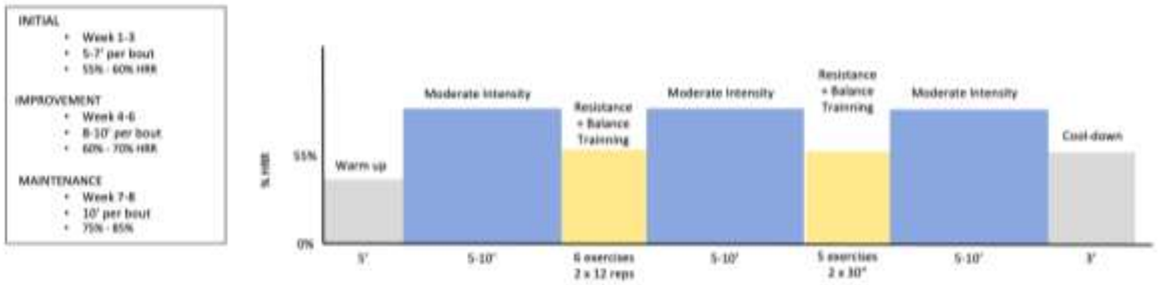

Supplement 2: Example of a multicomponent sprint interval training session

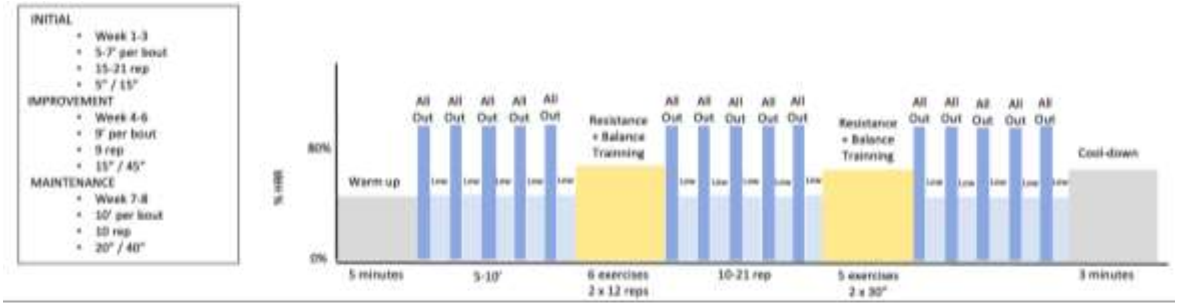

Supplement 3: Comparison between attained and targeted heart rate reserve within exercise delivery modes and multicomponent intensity training regimes

		**Home-based**			
		**HRR (%)**	**HRR target (%)**	**HR (bpm)**	**HR target (bpm)**
**Phase 1**	**SIT**	81±4	≥90	124±7	156 - 165
	**CAT**	72±5	55 - 60	117±1	116 - 119
**Phase 2**	**SIT**	82±2	≥90	128±8	156 - 165
	**CAT**	71±11	60 - 70	105±22	119 - 126
**Phase 3**	**SIT**	84±11	≥90	126±13	156 - 165
	**CAT**	67±11	75 - 85	92±22	130 - 137
		**Gym-based**			

		HRR (%)	HRR target (%)	HR (bpm)	HR target (bpm)
**Phase 1**	**SIT**	83±6	≥90	129±12	156 - 165
	**CAT**	75±9	55 – 60	105±22	116 - 119
**Phase 2**	**SIT**	76±10	≥90	124±15	156 - 165
	**CAT**	72±9	60 – 70	96±14	119 - 126
**Phase 3**	**SIT**	80±12	≥90	129±16	156 - 165
	**CAT**	72±8	75 - 85	100±16	130 - 137

Data are presented as mean ± standard deviation. Abbreviatures: HR = heart rate; HRR = heart rate reserve; SIT = sprint interval training; CAT = continuous aerobic exercise training.

Supplement 4: Comparison of body composition variables among delivery exercise modes and multicomponent intensity training regimes

**Anthropometry**		**SIT**	**CAT**	**Main effect of time (p-value; partial eta square)**	**Main effect of** multicomponent intensity training regimes **(p-value; partial eta square)**	**Interaction (p-value; partial eta square)**
Weight				(*p* = 0.20; η^2 = 0.11)	(*p* = 0.76; η^2 = 0.00)	(*p* = 0.40; η^2 = 0.07)
(kg)	**M1**	68.6±16.5	71.3±14.2	-	-	-
	**M2**	69.0±17.3	71.8±15.2	-	-	-
	**M3**	68.0±18.8	70.9±17.6	-	-	-
	**M4**	68.8±18.2	70.1±17.9	-	-	-
BMI				(*p* = 0.23; η^2 = 0.10)	(*p* = 0.85; η^2 = 0.00)	(*p* = 0.58; η^2 = 0.05)
(kg/m^2)	**M1**	26±4	27±6	-	-	-
	**M2**	27±5	27±6	-	-	-
	**M3**	26±5	27±7	-	-	-
	**M4**	27±5	27±8	-	-	-
WC				(*p* = 0.13; η^2 = 0.08)	(*p* = 0.87; η^2 = 0.00)	(*p* = 0.07; η^2 = 0.16)
(cm)	**M1**	0.89±0.10	0.93±0.12	-	-	-
	**M2**	0.92±0.17	0.99±0.22	-	-	-
	**M3**	0.91±0.18	0.92±0.15	-	-	-
	**M4**	0.92±0.17	0.75±0.32	-	-	-
Fat-Free Mass				(*p* = 0.37; η^2 = 0.02)	(*p* = 0.59; η^2 = 0.02)	(*p* = 0.62; η^2 = 0.04)
(kg)	**M1**	48.9±8.9	46.8±6.9	-	-	-
	**M2**	48.6±8.7	46.8±7.3	-	-	-
	**M3**	48.7±9.0	47±8.0	-	-	-
	**M4**	49.7±9.3	47±8.7	-	-	-
Fat mass				(*p* = 0.02; η^2 = 0.23)	(*p* = 0.44; η^2 = 0.04)	(*p* = 0.83; η^2 = 0.02)
(kg)	**M1**	19.7±9.8	24.6±8.3	-	-	-
	**M2**	20.3±10.6	25.1±9.5	M2 > M4	-	-
	**M3**	19.3±11.2	23.8±12.4	-	-	-
	**M4**	19.1±11.7	23.1±12.9	-	-	-

Data are presented as mean ± standard deviation. Abbreviations: BMI = body mass index; WC = waist circumference; SIT = sprint interval training; CAT = continuous aerobic exercise training; M = moment.
